# Efficacy and safety of the water pressure method for endoscopic submucosal dissection in superficial pharyngeal cancer

**DOI:** 10.1055/a-2284-9184

**Published:** 2024-04-26

**Authors:** Noriko Matsuura, Motohiko Kato, Kentaro Iwata, Kurato Miyazaki, Teppei Masunaga, Yoko Kubosawa, Mari Mizutani, Yukie Hayashi, Motoki Sasaki, Tomohisa Sujino, Kaoru Takabayashi, Teppei Akimoto, Yusaku Takatori, Atsushi Nakayama, Mariko Sekimizu, Hiroyuki Ozawa, Naohisa Yahagi

**Affiliations:** 1 38084 Division of Research and Development for Minimally Invasive Treatment, Cancer Center, Keio University School of Medicine, Shinjuku-ku, Japan; 2 Center for Diagnostic and Therapeutic Endoscopy, Keio University School of Medicine, Shinjuku-ku, Japan; 3 38084 Division of Gastroenterology and Hepatology, Department of Internal Medicine, Keio University School of Medicine, Shinjuku-ku, Japan; 4 38084 Department of Otolaryngology, Head and Neck Surgery, Keio University School of Medicine, Shinjuku-ku, Japan

**Keywords:** Diagnosis and imaging (inc chromoendoscopy, NBI, iSCAN, FICE, CLE), Endoscopy Upper GI Tract, Endoscopic resection (ESD, EMRc, ...), Pharyngeal cancer

## Abstract

**Background and study aims** Superficial pharyngeal cancers can be cured with transoral surgery (TOS), which preserves organ function and quality of life. Pharyngeal endoscopic submucosal dissection (ESD) is challenging to perform because of limited maneuverability and complex anatomical features. The water pressure method (WPM) is useful for natural traction techniques during ESD and is potentially useful for pharyngeal ESD. This study aimed to investigate the short-term outcomes of WPM-ESD for pharyngeal lesions.

**Patients and methods** Therapeutic outcomes of patients who underwent WPM-ESD for pharyngeal lesions at Keio University between May 2019 and February 2022 were retrospectively analyzed.

**Results** Twenty-one pharyngeal lesions treated with WPM-ESD were analyzed. Three lesions were located in the oropharynx and 18 in the hypopharynx. All ESD procedures were performed under general anesthesia. The endoscopic en bloc resection rate was 100%. The median procedure time was 15 minutes (range 4–45 minutes). All patients were successfully extubated on the day of ESD. No serious adverse events (AEs) related to WPM-ESDs were observed. None of the patients required nasogastric intubation, percutaneous endoscopic gastrostomy, or tracheotomy. The median fasting time and hospital stay were 2 days (range 2–5 days) and 6 days (range 6–10 days), respectively. All the histological results indicated squamous cell carcinoma. The complete histologic resection rate was 76.2%.

**Conclusions** WPM-ESD achieved a high en bloc resection rate and short procedure time without serious AEs. Thus, it may be a useful treatment for pharyngeal lesions.

## Introduction

Pharyngeal cancer occurs in approximately 136,000 individuals annually, worldwide, and incidence is increasing in developed countries 1 2 . The disease is often detected at an advanced stage because endoscopic observation is challenging owing to patient reflexes and poor prognosis 3 4 .

Recent developments in narrowband imaging (NBI) 5 and the establishment of a precise pharyngeal observation method 6 have enabled increased early-stage detection of superficial pharyngeal cancers. Early-stage pharyngeal cancers can be cured via transoral surgery (TOS), which preserves organ function and quality of life of the patient, and is an effective minimally invasive treatment 7 8 9 10 11 12 13 . TOS includes transoral video-assisted surgery, endoscopic laryngopharyngeal surgery, and endoscopic resection. The primary clinical advantage of TOS is that it protects patients who are candidates for organ- and function-sparing procedures from the potentially adverse events (AEs0 of radical surgery or chemoradiation. En bloc resection rates using endoscopic submucosal dissection (ESD) for pharyngeal cancer are high (77.4%–100%) in TOS 7 8 11 12 13 . The fasting period for ESD is typically 2 days. Endoscopic resection is considered less invasive among TOS.

Although ESD is associated with a higher complete resection rate (en bloc with negative histological resection margins) than endoscopic mucosal resection (EMR) and is a potentially therapeutic option, pharyngeal ESD is technically difficult because maneuverability in the pharynx is limited. In addition, approaching the lesion is difficult and visibility of the subepithelial layer during dissection is poor owing to the complex anatomical features of the pharynx.

Since Binmoeller et al. demonstrated the safety and efficacy of underwater EMR, the fluid immersion technique has been applied to ESD throughout the gastrointestinal tract 14 15 . Recently, ESD using the water pressure method (WPM-ESD) has been effective for gastrointestinal ESD as a natural traction method that reduces AEs and shortens procedure time 16 17 18 19 20 . The buoyant force of water immersion and the pumping pressure of the water jet aid in penetrating the submucosal layer without the need for mechanical traction. It is believed that these advantages of the WPM are applicable to pharyngeal lesions. However, the effectiveness of WPM-ESD for pharyngeal lesions has only been documented in a single case report 21 . Therefore, this study aimed to investigate the therapeutic outcomes and safety of WPM-ESD for treatment of pharyngeal lesions.

## Patients and methods

### Study participants

This retrospective observational study was conducted at the endoscopy unit of a Japanese referral university hospital using a database. Patients who underwent pharyngeal WPM-ESD between May 2018 and February 2021 were retrospectively analyzed. During this period, pharyngeal ESD was performed on 27 lesions. Of these lesions, WPM-ESD was performed on 21 lesions; the use of WPM was determined by videos at the time of treatment. In pharyngeal ESD, the indication for WPM-ESD was not defined by tumor site or diameter. WPM-ESD in pharyngeal ESD was not reported at the time of its introduction, and it was performed at the decision of the endoscopist, if deemed feasible. This study was conducted in according to the tenets of the 2008 Declaration of Helsinki. The study protocol was approved by the Institutional Review Board of the host hospital (20180163 and 20190139).

### Indication for ESD

Pharyngeal ESD is recommended for lesions that are suspected to be pharyngeal cancer based on endoscopic observations and histological findings. Diagnostic endoscopy using was used to identify indications for ESD (GIF-H260Z, 290Z, or 1200EZ; Olympus Medical Systems Co., Tokyo, Japan, or EG-L600ZW7; FUJIFILM, Tokyo, Japan). If the lesion exhibited a well-demarcated brownish area and irregular microvascular patterns on NBI, it was diagnosed as endoscopically suspected “superficial cancer.” The details of these findings have been previously reported 22 23 . The horizontal extent of the lesions was assessed using the Valsalva method as required 24 . The feasibility of pharyngeal ESD was determined through discussion between the otolaryngologists and gastroenterologists.

### ESD procedures

The ESD procedure was initiated with the patient under general anesthesia, and a laryngoscope was inserted into the supraglottis by an otolaryngologist. A laryngoscope was used to expand the working space as described previously 13 . ESD procedures were performed using an endoscope with a waterjet function (GIF-H290T or GIF-Q260J; Olympus Medical Systems, Co., Ltd.). A 1.5-mm Dual Knife J (KD-655Q; Olympus Medical Systems) was used. A small-caliber-tip transparent hood (ST Hood or ST Hood short-type; DH-28CR, DH-28GR, or DH-33GR; FUJIFILM) was used to facilitate entry of the endoscopic devices into the submucosal layer. Magnifying endoscopy or a 0.75% iodine solution was used to delineate the tumor margin and marking dots were circumferentially placed outside the margin using the Dual Knife J. Sodium thiosulfate solution (STS) was used to neutralize the iodine before ESD. For the submucosal injection, a 10% glycerine (Glycerol; Chugai Pharmaceutical Co., Ltd., Tokyo, Japan) or hyaluronic acid solution (MucoUp; Boston Scientific Japan, Tokyo, Japan) with a small amount of indigo carmine was used. A standard high-frequency generator (VIO3; ERBE Elektromedizin, Tubingen, Germany) and carbon dioxide insufflator (UCR; Olympus Medical Systems) were used. Four modes of electrosurgical currency were applied: “dry cut” (effect 2.2) or “endo cut” (effect 1.2) for mucosal incision and “swift coagulation” (effect 3.5) for submucosal dissection, “spray coagulation” (effect 1.2) for haemostasis using the tip of the knife, and “soft coagulation” (effect 3.0) for haemostasis using hemostatic forceps (Coagrasper, Olympus Medical Systems) ( Table 1 ). All procedures were performed by three experienced endoscopists who had performed >250 ESDs. Dexamethasone was intravenously injected immediately after removing the intubation tube to prevent laryngeal edema, as appropriate in consultation with the otolaryngologist. Dexamethasone was not used routinely. No difference in the use of dexamethasone or other perioperative management was observed between WPM-ESD and conventional ESD.

**Table:** **Table 1** Settings for the water pressure method after endoscopic submucosal dissection (WPM-ESD).

Mucosal incision	Dry cut (effect 2.2) End cut I (effect 1.0, duration 2.0, interval 2.0)
Submucosal dissection	Swift coagulation (effect 3.5)
Hemostasis using a knife tip	Spray coagulation (effect 1.2)
Pre-coagulation	Forced coagulation (effect 0.3)
Hemostasis using haemostatic forceps	Soft coagulation (effect 3.0)

The day after ESD, blood tests and chest radiography were performed to monitor any potential complications related to the procedure. Thereafter, the patient was allowed to start drinking water. Diet began 2 days after ESD. If the patient had sore throat or pain during swallowing, nonsteroidal anti-inflammatory drugs (NSAIDS) were administered as appropriate. The patient was discharged 4 days after ESD without any major AEs. Follow-up was conducted at our hospital within 1 month post-ESD for assessment of postoperative complications and histological assessments.

### WPM

Procedures in this trial. A transparent hood was placed and the submucosal injection administered. Thereafter, a whole circumferential incision was made, followed by dissection of the subepithelial layer to penetrate below the lesion with the water pressure method. The procedure time was 25 minutes.Video 1

**Fig. 1:**
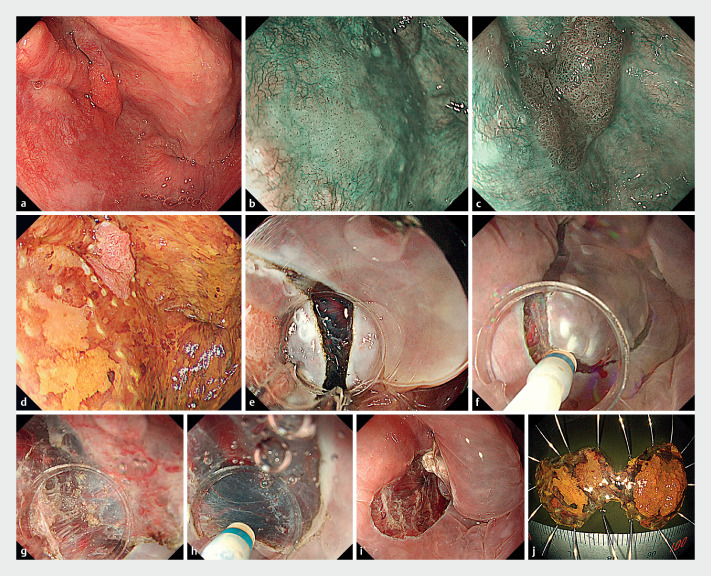
Endoscopic images of the water pressure method (WPM) method. **a** Pharyngeal observation in general anesthesia. **b** Type 0-IIa flat lesion located on the posterior wall of the hypopharynx. **c** Type 0-I lesion located on the left pyriform sinus. **d** A marking dot is placed outside of the lesion. **e** Incision was made from the anal side. **f** A whole circumferential incision was placed. **g** Dissection of the subepithelial layer using the water pressure method for the posterior wall of the hypopharynx. **h** Dissection of subepithelial layer with water pressure method for left pyriform sinus. **i** Two lesions were removed en bloc. **j** The pathological diagnosis was squamous cell carcinoma pTis for the posterior wall lesion of the hypopharynx, and pT1 tumor thickness was 1300μm for the left pyriform sinus lesion.

We have used WPM for duodenal ESD since June 2017. Subsequently, our pharyngeal ESD procedure shifted from conventional ESD to WPM-ESD since its introduction in May 2019. In WPM-ESD, the pharyngeal lumen is filled with saline solution. First, a circumferential incision was made. Thereafter, the space below the mucosal flap was opened using a water stream from the waterjet function of the endoscope ( Fig. 1 ). The pumping pressure of the water jet improves the lateral edge visualization of the dissection layer in the dissection plane. We infused the saline solution using an endoscopic flushing pump (OFP; Olympus Europe, Hamburg, Germany; Video 1 ). In pharyngeal ESD, arterial bleeding is rare and its incidence differs from the other organ ESD. In addition, pre-coagulation with forced coagulation mode is effective in inhibiting bleeding. If venous oozing occurs, identifying the bleeding point is easier in underwater situations, because the bleeding point in saline solution can be easily recognized.

### Histological assessment after ESD and ESD-related adverse events

The resected specimens were extended on a board with pins and fixed in 10% formalin for 24 hours. The preserved specimens were cut into consecutive 2-mm sections and examined by pathologists. The lesions were histologically classified as carcinoma in situ or subepithelial invasion. In cases of subepithelial invasion, tumor thickness was measured (the distance between the surface layer and the deepest point). Tumor size, invasion depth, lymphatic and vascular involvement, and tumor involvement in the lateral and vertical margins were assessed according to the Japanese guidelines for head and neck cancer 25 . R0 resection was defined as en bloc resection with negative horizontal and vertical margins. Furthermore, the horizontal margin (HM) was also investigated: HM0 was defined as no tumor exposed on any horizontal margin, HM1 as a tumor present on any vertical margin, and HMX as the inability to assess the existence of a residual tumor on the horizontal margin. The frequency of AEs, such as postoperative bleeding, perforation, laryngeal edema requiring prophylactic or emergency temporary tracheotomy, subcutaneous emphysema, aspiration pneumonia, fistula formation, abscess formation, and stricture formation, were evaluated.

### Measured outcomes

This study aimed to investigate short-term outcomes of pharyngeal WPM-ESD. They included en bloc resection, en bloc with R0 resection, procedure time, and complications. The primary outcome of this study was the endoscopic en bloc resection rate. The secondary outcomes of this study were: 1) ESD procedure time, which is defined as duration starting from initial dissection to completion of resection; 2) pathological results, including R0 resection; 3) fasting period; 4) hospital duration; and 5) AEs. All continuous and categorical variables are presented as median (range) and frequencies (percentages), respectively.

## Results

### Baseline characteristics

During the study period, 21 patients underwent pharyngeal ESD using the WPM. Baseline characteristics of the patients and their lesions are listed in Table 2 . The median age of patients was 67 years (range, 58–84). The patients comprised eight men (80%) and two women (20%). Fourteen percent of the lesions were located in the oropharynx and 86% in the hypopharynx. The most common macroscopic type was flat lesions (81%). The median endoscopic tumor diameter was 10 mm (range, 5–30 mm).

**Table:** **Table 2** Baseline characteristics of patients and lesions.

Total number of patients/lesions	10/21
Male, n (%)	8 (80)
Age, y, median (range)	67 (58–84)
History of esophageal cancer, n (%)	
Endoscopic treatment	6 (60)
Surgery	1 (10)
History of head and neck cancer, n (%)	
Endoscopic treatment	1 (10)
CRT	2 (20)
Location, n (%)	
Oropharynx	
posterior wall	3 (14)
Hypopharynx	
Pyriform sinus	9 (43)
Posterior wall	7 (33)
Post-cricoid	1 (5)
Side wall	1 (5)
Macroscopic type, n (%)	
Protruded	4 (19)
Flat	17 (81)
Tumor size (mm), median, (range)	10 (5–30)
CRT, chemoradiotherapy.	

### Therapeutic outcomes

Table 3 lists treatment-related outcomes. The endoscopic en bloc resection rate was 100%. The median procedure time was 15 minutes (range, 4–45 minutes). The median fasting period was 2 days (range, 2–5 days). The median hospital duration was 6 days (range, 6–10 days).

**Table:** **Table 3** Treatment-related outcomes.*

Lesions according to operator experience Experts n (%)	21 (100)
Procedure time, median (range) minutes	15 (4–45)
En bloc resection, n (%)	21 (100)
Fasting period, median (range) (day)	2 (2–5)
Hospital stay, median (range) (day)	6 (6–10)
Delayed bleeding, n	0
Extubation on day of ESD, n (%)	10 (100)
Tracheotomy, temporary, n	0
Aspiration pneumonia, n	0
Subcutaneous emphysema, n (%)	1 (10)
Nasogastric tube feeding, n	0
*Experts: Endoscopists who had performed >250 ESDs. ESD, endoscopic submucosal dissection.	

No cases involved use of a traction device, including the clip and thread technique. All patients were successfully extubated on the day of ESD and none required tracheotomy. One case of subcutaneous emphysema was observed in the hypopharyngeal lesion. No patients required nasogastric tube feeding or percutaneous endoscopic gastrostomy. No cases of laryngeal edema requiring temporary tracheotomy were observed. No cases of postoperative bleeding or aspiration pneumonia were observed. None of the remaining patients had any AEs other than sore throat or pain during swallowing, which were managed with NSAIDS for several days. All patients had preserved larynx, swallowing, speech, and airway functions. One case of prolonged fasting resulting from subcutaneous emphysema and another of nasal pain, potentially caused by irrigation of the nasal cavity with iodine-containing water, were noted. All patients were treated conservatively. No treatment-related death occurred during the study period.

### Histological results

Table 4 lists the histological results. All lesions were squamous cell carcinoma (SCC). The T categories were Tis (six lesions, 29%), T1 (12 lesions, 57%), and T2 (three lesions, 14%). Fifteen lesions were subepithelial SCC. The median tumor thickness for subepithelial lesions was 200 μm (range, 100–1300 μm). The pathologically R0 resection rate was 76.2% (16 lesions). Of the five patients with lesions undergoing follow-up with HMX or HM1, four underwent follow-up endoscopy. No recurrence was observed (median surveillance period, 461 days [range, 330–1147 days]).

**Table:** **Table 4** Histological result.

Pathological findings, n (%) Squamous cell carcinoma	21 (100)
Invasion depth, n (%)	
Intraepithelial/subepithelial	6 (28.6)/15 (71.4)
Tumor thickness for subepithelial lesions, median (range) (μm)	150 (100–800)
T category, n (%)	
Tis/T1/T2	6 (28.6)/12 (57.1)/3 (14.3)
Lymphatic invasion	0
Venous invasion	0
Margin status, n (%)	
Horizontal margin negative (HM1)	16 (76.2)
Horizontal margin unclear (HMX)	2 (9.5)
Horizontal margin positive (HM0)	3 (14.3)
Vertical margin negative (VM0)	21 (100)
Vertical margin positive (VM1)	0 (0)
R0 resection, n (%)	16 (76.2)
HM0, pathological horizontal margin negative; HMX, pathological horizontal margin unclear; HM1, pathological horizontal margin positive; VM0, pathological vertical margin negative; VM1, pathological vertical margin positive.	

## Discussion

This study focused on technical aspects of WPM-ESD for superficial pharyngeal cancer and demonstrated its short-term outcomes. In this study, all lesions were successfully en bloc resected and the median procedure time was 15 minutes, which was shorter than that reported in previous studies of conventional pharyngeal ESD. The median hospital stay was 6 days without serious AEs, including laryngeal edema.

Although ESD is a good treatment option, it is technically challenging owing to the anatomical features of the pharynx. The technical difficulty of pharyngeal ESD is strongly affected by the accessibility of the target lesion, availability of adequate maneuverable space, and mutual interference between the endoscope, laryngoscope, and intubation tube. Performing ESD on lesions located in the pyriform sinus poses technical challenges, owing to the narrow and intricate space that makes accessing the lesion difficult. ESD of lesions located on the posterior walls of the oropharynx and hypopharynx is challenging because of the shallow subepithelial layers. The median procedure time for pharyngeal ESD is 50 to 124.9 minutes 11 12 13 . To overcome this technical difficulty, several mechanical traction techniques, including the use of laryngeal forceps, clip and thread technique, ring-shaped thread traction, and the grasping forceps method, have been reported 26 27 28 . However, occasionally, the direction of traction cannot be controlled, and the forceps interfere with the endoscope. Effective traction is not always achieved.

To overcome these difficulties, WPM-ESD is considered effective for pharyngeal ESD. WPM-ESD is performed in underwater conditions; hence, capitalizing on its features (floating and magnified effects) is advantageous. The “floating” effect, which works opposite to the direction of gravity, can elevate the lesion during underwater submucosal dissection. Endoscopists usually position the patient such that the lesion is in the direction of gravity. Under underwater conditions, the lesion is submerged, and endoscopists do not need to consider the issue of gravity. This is particularly useful for post-cricoid lesions. Lesions in the post-cricoid region, which are often anatomically limited vertically, can be easily dissected using the floating effect. In addition, the magnification effect precisely aids visualization of the dissecting point. The underwater image is magnified approximately 1.33 times owing to the refractive index of water, and the optical zoom effect enables a more precise procedure 29 , even if hemorrhaging occurs. The bleeding site is well visualized underwater 30 . Consequently, endoscopists can dissect the subepithelial layer more precisely.

In addition, the pumping pressure of the water jet facilitates ESD. The advantages of the WPM-ESD are as follows: 1) simple and easy, without special equipment or devices; 2) ability to create the mucosal flap within a confined space (natural traction); 3) ease of locating the edge of the lesion; and 4) ability to effectively address fibrotic lesions near scars or those located on the posterior wall. The strengths of this study are as follows. First, the WPM-ESD technique is versatile and can be applied at any point during the procedure. To use it, you simply need to activate the foot pedals connected to the endoscope pump and initiate the water irrigation. Although several mechanical traction techniques have been reported, most of them require special devices or equipment. Second, WPM-ESD enables the surgeon to move below the mucosal flap. In the early stages of dissection, the mucosal flap is relatively small; therefore, encountering the subepithelial space is difficult, in addition to the complex anatomical features of the pharynx. Using the ST hood instead of the conventional transparent hood allows better visualization for penetrating the subepithelial layer and better traction. Third, water irrigation makes it easier to locate the edge of the lesion. WPM-ESD allows safe dissection of the lateral edge from the outside to the inside of the lesion (pull-off method), which is typically impossible with a tipped or Dual Knife. This pull-off method is beneficial for pharyngeal ESD because of the limited working space and difficulty in approaching the lesion owing to its complex anatomical features. Fourth, using a water stream allows easier visualization of the dissection line, especially for lesions with severe fibrosis near a previous ESD scar or in the oropharyngeal posterior wall, where the subepithelial layer is shallow. Subepithelial, muscularis, and fibrotic tissues can be distinguished based on the degree of tissue vibration when an active water stream is applied. Owing to the advantages of WPM, no cases of postoperative laryngeal edema or aspiration pneumonia were found, as the treatment could be efficiently completed in a short duration.

In WPM-ESDs, a saline solution is preferable for the following reasons: First, a saline solution exhibits increased buoyancy compared with water due to its higher specific gravity. Second, the risk of electrolyte imbalance is lower when using saline solutions than when using water alone. Third, the presence of electrolytes in saline solutions provides superior electrical conductivity under fluid immersion conditions and facilitates clean tissue cutting 31 .

One disadvantage of WPM-ESD is that bleeding can lead to cloudiness in the water, causing impaired visibility. Arterial bleeding is rare in pharyngeal ESD compared with that in gastric or duodenal ESD. If vessels are visible during ESD, detailed cauterization and resection with preforced coagulation can prevent intraoperative bleeding. If intraoperative bleeding causes cloudy water and poor vision, water in the pharyngeal space must be appropriately aspirated. Another disadvantage of WPM-ESD for pharyngeal lesions is the risk of aspiration pneumonia. In our retrospective trial, aspiration pneumonia was not observed in any patient. This may be because the cuff on the intubation tube prevented water from flowing into the trachea. There is also a risk of iodine reflux into the nasal cavity due to water irrigation in the pharyngeal space. In this study, one case of post-ESD nasal pain, possibly caused by iodine, was observed. To overcome this, STS was sprayed to neutralize the iodine before initiating ESD. This process may inhibit the iodine content of the irrigating water.

If the lesion is resected with a negative margin, no additional treatments are generally performed and the patient is in the surveillance period for oropharyngeal and hypopharyngeal lesions. In head and neck lesions, no correlation was established between the histological features and lymph node metastasis. The “resect and watch” strategy, which involves local resection and observation until local lesion recurrence, seems feasible for functional or organ preservation 32 . Before the ESD procedure, all treatment strategies were discussed with the otolaryngologists, and during the ESD procedure, they were executed with collaborative efforts, which are essential for a safe ESD. We first showed that WPM-ESD for pharyngeal lesions achieved a high en bloc resection rate and short procedure time without serious adverse events.

This study had some limitations. First, it was a retrospective trial performed at a single university hospital. We did not compare the short-term therapeutic outcomes with conventional pharyngeal ESD or the mechanical traction method. Therefore, it would be beneficial to perform a prospective study to investigate the efficacy of WPM-ESD. We are currently conducting a prospective trial (UMIN000047207) to investigate the short-term outcomes of pharyngeal ESD. Second, all procedures were performed by experienced endoscopists familiar with ESD of the gastrointestinal tract, such as the esophagus, stomach, and colon. However, after pharyngeal ESD, several postoperative AEs, including laryngeal edema and subcutaneous emphysema, can be critical, and this procedure is better performed by endoscopists who specialized in the pharyngeal field. Third, the amount of water irrigated during the WPM-ESD was not measured. We intend to investigate these issues in a prospective trial.

## Conclusions

WPM-ESD for pharyngeal lesions achieved a high en bloc resection rate and short procedure time without serious AEs. Thus, it may be a useful treatment method for the natural traction of pharyngeal lesions.
